# Revealing the potential of *Gastrodia elata* as a therapeutic candidate for degenerative diseases

**DOI:** 10.1016/j.jgeb.2026.100707

**Published:** 2026-06-02

**Authors:** Jia Jun Chuah, Morris Jehssica Shri, Balakrishnan Shanthakumar, Krishnamoorthy Venkateskumar, Karupiah Sundram, Subramani Parasuraman

**Affiliations:** a Pharmacology, Toxicology, and Basic Health Sciences Unit, Faculty of Pharmacy, AIMST University, 08100 Bedong, Kedah, Malaysia; b Department of Pharmaceutical Chemistry, Faculty of Medicine and Health Sciences, SRM College of Pharmacy, SRMIST, Kattankulathur, Tamil Nadu 603203, India; c Pharmaceutical Technology Unit, Faculty of Pharmacy, AIMST University, 08100 Bedong, Kedah, Malaysia; d Pharmaceutical Chemistry Unit, Faculty of Pharmacy, AIMST University, 08100 Bedong, Kedah, Malaysia

**Keywords:** *Gastrodia elata*, Gastrodin, Molecular docking, Antioxidant

## Abstract

**Background:**

*Gastrodia elata* is commonly used in Eastern traditional medicine, mainly for disorders and ailments related to the central nervous system. The main active phytochemical compound is gastrodin, and it has been shown to possess many neuroprotective properties. There is a lack of systematic correlation between phytochemical profiling and computational modeling. Hence, the present study was planned to investigate the phytochemical profiling of the rhizome of *G. elata*, evaluate its antioxidant potential, conduct molecular docking studies against biological targets related to oxidative stress and neurodegeneration, prediction of pharmacokinetic properties of gastrodin and understanding the link between phytochemical composition and pharmacological mechanisms.

**Materials and methods:**

Powdered of dried *Gastrodia elata* rhizomes was extracted with ethanol using the hot percolation method. A confirmatory quantitative analysis was performed to determine the concentration of phenolic and flavonoid compounds present. Gas chromatography-mass spectrometry analysis was conducted to identify the compounds present in the extract. FTIR spectral data of the ethanolic extract of *Gastrodia elata* (EEGE) were obtained using a Fourier transform infrared instrument and compared with a reference standard to determine the functional groups present. The antioxidant capacity in EEGE were identified using the DPPH radical scavenging method. The main active compound of *Gastrodia elata*, is gastrodin. The canonical simplified molecular-input line-entry system (SMILES) format is used for biological activity prediction via online platforms. Molecular docking of gastrodin was performed using Auto Dock version 4.2. Pharmacokinetic and toxicological activities for gastrodin were conducted using the online web tools pkCSM and ProTox 3.0.

**Results:**

The EEGE showed the presence of alkaloids, saponins, flavonoids, glycosides, terpenoids and phenolic compounds, but the absence of steroid compounds. GC–MS analysis showed that ethane, 1,1-diethoxy- presented the highest probability and peak percentage. FTIR spectral analysis showed similarities and differences in the chemical profiles between the gastrodin standard and EEGE. Results showed both EEGE and gastrodin standard exhibited absorption peaks at 3222.33 cm^−1^ (hydroxyl stretch), supporting the presence of gastrodin in the ethanolic extract. PASS prediction analysis for EEGE’s bioactive compound, gastrodin, showed a wide range of potential pharmacological activities with high probability scores (Pa > 0.6). The highest predicted probabilities were vasoprotective, anti-infective, cholesterol antagonistic and cardioprotective activities.

**Conclusion:**

This study confirms the presence of gastrodin as the major bioactive compound from *Gastrodia elata* rhizomes and attributes strong antioxidant and neuroprotective potential to it. Integrated phytochemical, *in silico*, and pharmacokinetic analyses support its traditional use in CNS-related disorders and highlight gastrodin as a promising and safe therapeutic candidate for oxidative stress and neurodegenerative conditions.

## 1 Introduction

The practice of traditional medicine in Eastern countries, particularly the use of plant-based remedies for the treatment of various ailments, remains widespread and scientifically relevant. Several medicinal herbs, including *Ginkgo biloba* are well known for their neuroprotective flavonoids and terpenoids that enhance cognitive function and mitigate oxidative stress. *Withania somnifera* (Ashwagandha) exhibits anti-neurodegenerative effects through its withanolides, which modulate neuroinflammation and apoptotic pathways. *Curcuma longa* (turmeric), rich in curcumin, has demonstrated significant antioxidant and anti-amyloidogenic properties relevant to neurodegenerative disorders such as Alzheimer’s disease. In this context, *Gastrodia elata* Blume (commonly known as Tianma, a traditional Chinese herb) has been used to treat neurological ailments such as headaches, convulsions, stroke, epilepsy, insomnia and dizziness for many years.^1^ It has also been shown to possess neuroprotective, anti-inflammatory, anti-angiogenic, cardioprotective and antidepressant properties.^2^ The water extract of *G. elata* exhibited no genotoxic or oral toxicity after 28 days of administration.^3^ In addition, pretreatment with *G. elata* in rats with acetaminophen-induced toxicity in the liver and kidney toxicity demonstrated protective effects and minimal damage to the respective organs.^4^ Additionally, *G. elata* is combined with other herbs such as *Panax Ginseng C.A. Mey* (ginseng), *Euonymus alatus (Thunb.) Sieb*, and *Ligusticum chuanxiong Hort* to form Shen Ma Yi Zhi granule (SMYZG), a prescription for treating vascular disease. The aqueous extract of SMYZG has been shown to possess neuroprotective benefits and improve learning and memory, affecting the hippocampal structure and function of the central cholinergic system. Based on the 2-VO and MID models, it has been shown to suppress the inflammatory and oxidative stress responses.^5^ There are over 80 types of polysaccharides, organic acids, phenols and sterols that have been isolated from *G. elata*.^6^ Huang et al.^7^ showed that fresh *G. elata* was able to reduce cognitive decline caused by chronic restraint stress in mice.

Gastrodin, also known as 4-hydroxybenzyl alcohol-4-*O*-β-D-glucoside is isolated as the main active compound in *Gastrodia elata* Blume. Gastrodin is soluble in water and methanol but insoluble in ether and chloroform.^8^ It possesses neuroprotective capabilities, and is employed in the treatment of neurological disorders. This is evidenced by its ability to improve cognitive deterioration in mouse models of Alzheimer’s disease, Parkinson’s disease and vascular dementia. Gastrodin has shown positive improvement on 3,3′-iminodipropionitrile-induced memory impairments in rats by regulating the serotonergic system by altering the 5-HT, SERT and 5-HT1A receptor levels.^9^ It can also act as a protecting agent against apoptosis induced by methamphetamine.^10^ It has also been shown to possess capabilities in preventing osteonecrosis.^11^ Gastrodin can protect against cardiovascular diseases by protecting myocardial cells.^12^ Gastrodin has also been shown to ameliorate atherosclerosis by down-regulating the NF-κB pathway to inhibit the inflammatory response and formation of foam cells.^13^

Although *G. elata* has been traditionally used in Asian medicine for its neuroprotective, antioxidant, and anti-inflammatory properties, the comprehensive identification of its bioactive compounds and their molecular mechanisms remain unexplored. Previous studies have primarily focused on individual constituents or crude extracts without integrating *in silico* molecular docking analyses to elucidate their potential targets at the molecular level. Moreover, there is a lack of systematic correlation between phytochemical profiling and computational approaches that identify the specific mechanism responsible for its pharmacological activities. The present study aimed to investigate the phytochemical profiling of the rhizome of *G. elata* using analytical techniques like GC–MS and FTIR to identify bioactive constituents, evaluate the antioxidant potential and perform molecular docking studies against biological targets related to oxidative stress and neurodegeneration, as well as prediction of pharmacokinetic properties, in order to understand the correlation between phytochemical composition and pharmacological mechanisms.

## 2 Materials and methods

### 2.1 Materials

Dried *Gastrodia elata* rhizome was purchased from a Traditional Chinese Medicine (TCM) shop located in Georgetown, Penang. It was identified and verified by the TCM practitioner as the species *Gastrodia elata*. Using an electric blender, the dried rhizome was blended into a coarse powder.

### 2.2 Methods

#### 2.2.1 Preparation of *Gastrodia elata* rhizome for extraction

The dried rhizome powder was then extracted by using hot percolation with the help of a Soxhlet apparatus, using ethanol as the solvent at 80°C. The extraction process is completed once the resulting solvent turns transparent, which usually requires 3–4 cycles. The extracted concentrate is further concentrated by using a vacuum rotary evaporator (Yamato RE300) under a controlled temperature of 45°C–50°C. The final dry mass was weighed, and the percentage yield was calculated. Later, the extract was stored an amber glass container and kept refrigerated for further testing.

#### 2.2.2 Qualitative test

In order to identify the predominant classes of chemicals such as tannins, saponins, flavonoids, alkaloids, phenols, glycosides, steroids and terpenoids present in the ethanolic extract of *Gastrodia elata*, the extracts were subjected to confirmatory qualitative phytochemical analysis, using standard methods.^14^

#### 2.2.3 Qualitative analysis

The ethanolic extract of *Gastrodia elata* undergoes confirmatory quantitative analysis to determine the concentration of phenolic and flavonoid compounds in the sample using the method described elsewhere.^15^, ^16^, ^17^

#### 2.2.4 Gas Chromatography-Mass Spectrometry analysis

The analysis was conducted using the Perkin Elmer Clarus 600 GCMS equipped with TurboMatrix Headspace Sampler 40, and an Elite 5MS column (30.0 m × 250 µm × 0.25 µm). The oven temperature program for the GC was as follows: initial temperature set at 40°C and held for 2 min, followed by a ramp of 10°C per minute until reaching the final temperature of 200°C, where it was held for an additional 5 min. The carrier gas used was helium. The injection volume was set to 1 µL with a split ratio of 20:1, and the injector temperature was maintained at 200°C. A solvent delay of 2.00 min was implemented to prevent solvent interference. The transfer line temperature was set to 250°C, and the ion source temperature was maintained at 230°C. The mass spectrometer was operated in electron ionization (EI) mode with positive ion detection (Scan EI+), and the mass range scanned was from 50 to 500 Da. The Total Ion Chromatogram (TIC) was recorded, with the maximum ion current observed being 5.50e8. The compounds obtained were identified by comparing their mass spectra with those in the MAINLIB database, ensuring accurate characterization of the analytes present in the samples.

#### 2.2.5 Fourier-Transform Infrared Spectroscopy (FTIR)

The FTIR spectra of the ethanolic extract of *Gastrodia elata* were obtained using the FTIR instrument (Perkin Elmer Spectrum Two). The instrument operation and data processing were handled by PC-based software. A small amount of the dried extract was mixed with potassium bromide (KBr) and compressed to form pellets for the FTIR analysis, by applying pressure. Data on the infrared transmittance were obtained within the wavenumber range of 4000 cm^−1^–500 cm^−1^. The spectral data were compared to a reference in order to determine the functional groups that are present in the ethanolic extract sample.^18^

#### 2.2.6 Antioxidant level and capacity of *Gastrodia elata*

The ethanolic extract of *Gastrodia elata* was used in *in vitro* antioxidant assay to identify the level of antioxidants and the antioxidant capacity of the extract using the 2,2-diphenyl-1-picrylhydrazyl (DPPH) radicals scavenging method,^19^ hydroxyl radical scavenging method^20^ and the ferrous reducing antioxidant capacity assay.^21^

#### 2.2.7 Prediction of activity spectra for substances (PASS)

Gastrodin is the main active compound present in the rhizome of *Gastrodia elata*. The canonical simplified molecular-input line-entry system (SMILES) format of gastrodin was obtained from PubChem [https://pubchem.ncbi.nlm.nih.gov/] and used for biological activity prediction. The prediction of biological activity spectra was carried out with online prediction of activity spectra of substances (PASS) prediction tools (https://www.pharmaexpert.ru/passonline/).^22^ The input canonical SMILES format of gastrodin that was used for the prediction of the biological activity spectra is “C1=CC(=CC=C1CO)OC2C(C(C(C(O2)CO)O)O)O”.

### 2.3 Molecular docking

Molecular docking is a process of identifying the specific amino acid interactions that exist between the ligand and the target active site. A grid-based molecular docking approach was employed, where the target protein was defined using a receptor grid, followed by ligand docking using a genetic algorithm to evaluate binding affinity and interaction profiles. The docking validation was done by evaluating the RMSD value with the co-crystallised ligand for the target p38, which is the commonly associated with neurodegenerative disease. The superimposed structure resulted in the RMSD < 2 Å. The rationale for target protein selection is that the selected proteins are involved in inflammatory signalling pathways, oxidative stress regulation, apoptosis and neuronal survival. These pathways are involved in the neurodegenerative disorders, cancer, cardiovascular issues and inflammatory conditions. All these proteins belong to five major classes namely, ACE, which regulates blood pressure and cardiovascular inflammation; MAPK 14/p38, a mitogen-activated protein kinase pathway that mediates cellular response to stress, inflammatory signals, and cytokines. In addition, it regulates gene expression and apoptosis. BAX and BCL2 are apoptosis-regulating proteins that are vital in neurodegeneration and tissue damage. NRF2 protects the cell from reactive oxygen species. NGF is a neurotrophic signalling pathway that supports neuron survival, growth and repair.

The targets and their PDB codes were selected based on the Ramachandran plot available online in the Protein Data Bank database. Chemical structures of the compounds were drawn using ChemDraw version 8.0. Targeted proteins were prepared by using UCSF Chimera version 1.17.3. The protein preparation process includes optimization of the protein structure, removal of water, and addition of polar hydrogen atoms. A docking program was employed, where the receptor is fixed, and ligands were flexible, allowing exploration of the active site for binding. The proteins were assigned Kollman charges, and the grid box was generated around the target protein with x, y, and z dimensions of 126 * 126 * 126 Å with a spacing 1 Å.

The compound gastrodin was geometry optimized using Avogadro V 1.2.0. The primary aim of this approach is to refine the molecular structures to remove any strain and ensure a defined geometry. Molecular docking was performed using Autodock 4.2. The results obtained from docking were ranked based on their binding energy scores and corresponding conformations, which is likely to indicate the strength of binding. BIOVIA Discovery Studio Visualizer was used to visualize the docked complexes, as it enables to examination of both 2D and 3D interactions. The amino acid interactions between the ligands and target were analyzed and presented in a table together with the docking scores, number of interacting residues and the type of interaction involved. By predicting the potential binding affinities, this systematic approach is important for drug discovery and for understanding possible molecular mechanisms.

### 2.4 Absorption, Distribution, Metabolism, Excretion and Toxicity (ADMET) analysis

The molecular structure of gastrodin was obtained from PubChem (https://pubchem.ncbi.nlm.nih.gov), an open chemistry database. The Simplified Molecular Input Line Entry Specification (SMILES) of the compound was obtained from PubChem as well. An online Webtool named ProTox3.0 (https://tox.charite.de/protox3/) and pkCSM^23^ were used to obtain the absorption, distribution, metabolism, excretion and toxicity (ADMET) parameters.

## 3 Results

### 3.1 Qualitative and quantitative analysis

**Fig. 1:**
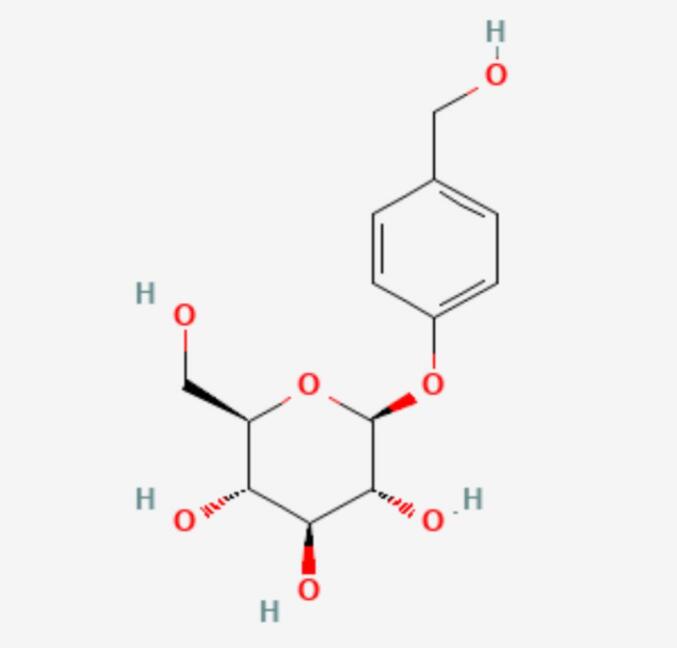
Molecular structure of gastrodin.

The percentage yield of the ethanolic extract of *Gastrodia elata* is 8.19% (w/w). Table 1 shows the name, identifiers, molecular fingerprints and basic properties of gastrodin, while Fig. 1 shows the molecular structure of gastrodin. The ethanolic extract obtained from the solvent extraction was subjected to qualitative phytochemical evaluation for the detection of the chemical constituents present in it. The ethanolic extract showed the presence of alkaloids, saponins, flavonoids, glycosides, terpenoids and phenolic compounds. The extract showed no presence of steroids.

**Table 1:** Name, identifiers, molecular fingerprints and basic properties of gastrodin.

Property	Value
Common name	Gastrodin
PubChem CID	115,067
Molar mass	286.28 g/mol
Exact mass	286.10525291 Da
Formula	C_13_H_18_O_7_
IUPAC name	(2R,3S,4S,5R,6S)-2-(hydroxymethyl)-6-[4-(hydroxymethyl)phenoxy]oxane-3,4,5-triol
SMILES	C1=CC(=CC=C1CO)O[C@H]2[C@@H]([C@H]([C@@H]([C@H](O2)CO)O)O)O
InChl	InChI = 1S/C13H18O7/c14-5–7-1–3-8(4–2-7)19–13-12(18)11(17)10(16)9(6–15)20–13/h1-4,9-18H,5-6H2/t9-,10-,11+,12-,13-/m1/s1
InChlKey	PUQSUZTXKPLAPR-UJPOAAIJSA-N
IUPAC condensed	Glc(b)-O-Ph(4-CH2OH)

### 3.2 Prediction of activity spectra for substances (PASS)

According to PASS prediction analysis, gastrodin has a wide range of possible pharmacological activities with high probability scores (Pa > 0.6). The highly expected results are vasoprotective and anti-infective properties, as well as cholesterol-antagonistic and cardioprotective capabilities. Gastrodin in particular showed potential neurological pathway regulation, specifically as a caspase stimulator and GABA aminotransferase inhibitor, therefore supporting neuroprotective and anti-apoptotic functions. Among the metabolic and organ-protective properties predicted are antihypercholesterolemic, hepatoprotective, antidiabetic, anti-hypoxic and antioxidant effects. Additionally, possible chemopreventive and anticancer properties, such as antineoplastic activity and caspase-mediated apoptotic regulation. The multifunctional therapeutic value of gastrodin is further demonstrated by additional anticipated outcomes such as immunostimulation, antiviral (influenza), antifungal, antithrombotic and inhibition of lipid peroxidation.

### 3.3 Gas Chromatography–Mass Spectrometry (GC–MS) analysis

The possible compounds present in the ethanolic extract of G. elata were predicted based on gas chromatography–mass spectrometry (GC–MS) spectra. The obtained data were analyzed, and the compounds with a relative probability greater than 30% are listed in Table 2.

**Table 2:** Gas chromatography–mass spectrometry analysis result of *Gastrodia elata* ethanolic extract.

S. No.	RT	Compound name	Probability	Molecular formula	MW	Area % peak
1	2.187	Ethane, 1,1-diethoxy-	89.1	C_6_H_14_O_2_	118.18	94.019
2	3.972	Butane, 1,1-diethoxy-	62.1	C_8_H_18_O_2_	146.23	1.54
3	3.972	Propane, 1,1-diethoxy-2-methyl-	35.7	C_8_H_18_O_2_	146.23	1.54
4	4.313	1-Butanol, 3-methyl-, acetate	77.7	C_7_H_14_O_2_	130.18	0.21
5	4.942	Pentanoic acid, 2-hydroxy-, ethyl ester	50.5	C_7_H_14_O_3_	146.18	0.002
6	5.197	Ethanol, 2-(2,3-butadienyloxy)-	61.8	C_5_H_10_O_3_	114.14	0.001
7	5.401	Butane, 1,1-diethoxy-3-methyl-	92.1	C_9_H_20_O_2_	160.25	0.88
8	6.676	Disulfide, methyl 1-methyl-1-(methylthio)propyl	82.8	C_5_H_12_S_3_	168.4	0.006
9	7.340	Propane, 1,1,3-triethoxy-	75.3	C_9_H_20_O_3_	176.25	0.086
10	8.802	2-Cyclohexen-1-one	57.3	C_6_H_8_O	96.123	0.023
11	8.802	But-1-ene-3-yne, 1-ethoxy-	33.0	C_6_H_8_O	96.13	0.023
12	12.033	4-Hepten-3-one, 5-methyl-	75.4	C_8_H_14_O	126.20	0.426
13	13.241	Sarcosine, n-propargyloxycarbonyl-,propargylester	67.5	C_12_H_19_NO_4_	241.28	0.003
14	14.023	á-D-Glucopyranose, 1,6-anhydro-	58.9	C_6_H_10_O_5_	162.14	0.052
15	14.499	Diethyl Phthalate	36.3	C_12_H_14_O_4_	222.24	0.311
16	17.713	1,2-Benzenedicarboxylic acid, mono(2-ethylhexyl)ester	42.7	C_24_H_38_O_4_	278.34	0.071
17	18.461	Hexadecanoic acid, ethyl ester	70.7	C_18_H_36_O_2_	284.48	1.499

### 3.4 Fourier transform-infrared spectroscopy analysis

Fourier-transform infrared spectroscopy (FTIR) was used to analyze the ethanolic extract of *G. elata* alongside pure gastrodin as the standard reference compound. The FTIR spectra showed distinct absorption peaks characteristic of functional groups present in the sample. Pure gastrodin exhibited prominent absorptions at 3222.33 cm^−1^ (hydroxyl groups), 2883.97 cm^−1^ (C–H stretching), and 1663.94 cm^−1^ (carbonyl stretch), with additional peaks in the ranges of 1600–1300 cm^−1^ and 1450–1050 cm^−1^. The ethanolic extract exhibited distinct absorptions at 3293.78 cm^−1^ (O–H stretching), 2928.10 cm^−1^ (C–H stretching), and between 1645.32 cm^−1^ and 1510.86 cm^−1^. The comparison between gastrodin standard and the ethanolic extract are shown on Table 3.

**Table 3:** FTIR analysis of standard gastrodin and ethanolic extract of gastrodin.

Sample	Absorption spectrum, wavenumber (cm^−1^)								
	3375–3260	2940–2925	1785–1640	1640–1600	1600–1300	1450–1050	1080–6200	935–710	600–400
**Standard pure gastrodin**	3222.33	2883.97	1663.94	1610.81	1511.16, 1465.05, 1396.76, 1368.17, 1343.95, 1301.64	1229.35, 1178.52, 1114.12, 1105.62, 1074.16, 1044.37, 1019.10	927.14, 904.30, 855.31, 841.94, 655.39, 623.79	829.24, 814.38, 778.16	575.46, 513.79, 483.30
**Ethanolic extract**	3293.78	2928.10	1645.32		1510.86, 1343.48, 1228.36	1148.31, 1102.38, 1074.42, 1011.64	854.97, 814.24		572.56, 514.18

### 3.5 Antioxidant level and capacity of *Gastrodia elata*

Three tests were conducted to assess the ability of the ethanolic extract of *G. elata* in neutralizing free radicals and inhibiting oxidative damage. IC_50_ results from DPPH assay on the ethanolic extract of *G. elata* with ascorbic acid as a control, hydroxyl radical scavenging assay and ferrous reducing antioxidant capacity assay are summarized in Table 4.

**Table 4:** IC_50_ of DPPH, hydroxyl radical scavenging assay and ferrous reducing antioxidant capacity assay of EEGE.

Test	IC_50_
2,2-diphenyl-1-picrylhydrazyl (DPPH) radical scavenging activity	723.61 ± 9.34
Hydroxyl radical scavenging assay	749.55 ± 36.19
Ferrous reducing antioxidant capacity assay	14.96 ± 2.04

### 3.6 Docking and *in silico* analysis

Molecular docking of gastrodin was conducted with proteins ACE, BAX, BCL2, CX3CR1, MAPK14, MAPK, nerve growth factor (NGF), NF-κB, NRF2, p38 and TNF-α.

The results of the docking of gastrodin against various PDB IDs were presented in Table 5, with their binding energies, which are expressed in kcal/mol and the type of amino acids and interaction types. Table 6 shows the 2D and 3D interactions of the proteins. The compounds exhibited different binding conformations with different energies, from which the top conformation was selected based on the binding affinity and scores were presented.

**Table 5:** Docking results and amino acid interactions.

SI.no.	Protein code	Docking score (Kcal/mole)	Amino acid and the type of interaction
1.	ACE	−3.6	PHE330, ARG289, PHE288, SER286 (Conventional hydrogen bonds)
2.	BAX	−2.82	GLU146, ARG147, GLY103, PHE105 (Conventional hydrogen bonds)
3.	BCL2	−1.72	ARG 207 (Conventional hydrogen bonds)MET206, PRO204, MET206, SER205 (Hydrogen bonds)VAL92 (Pi-alkyl)GLU200 (Pi-alkyl)
4.	CX3CR1	−2.68	LEU79, LEU151, ILEIII, ILE158 (Pi-alkyl)TRP154 (Pi-Pi)
5.	MAPK14	−2.65	ARG94, ARG5, VAL89 (Conventional hydrogen bonds)ILE346, TYR 342, THR 91, PHE90, VAL 345, PHE348, PRO 6, ALA93 (Hydrogen bonds)
6.	MAPK	−2.99	PHE34, CYS50 (Conventional hydrogen bonds)LYS52 (Pi-alkyl)
7.	Nerve Growth Factor (NGF)	−3.48	THR292, LEU290 (Van der Waals)
8.	NF-κB	−2.45	ARG366, GLU369 (Conventional hydrogen bonds)PRO370, PRO371 (Van der Waals)ARG368 (Pi-alkyl)
9.	NRF2	−4.28	ARG415, ARG380 SER363, ASN382 (Van der Waals)
10.	p38	−3.07	SER350, ASP325, GLN80 (Conventional hydrogen bonds)HIS78, LYS77 (Van der Waals)
11.	TNFALFA	−1.69	GLU49 (Van der Waals)ALA119 (Pi-alkyl)ASP47 (Conventional hydrogen bonds)

**Table 6:** 3D and 2D interaction of the proteins.

Protein code	3D interaction	2D interaction
ACE		
BAX		
BCL2		
CX3CR1		
MAPK14		
MAPK		
Nerve Growth Factor (NGF)		
NF-κB		
NRF2		
p38		
TNFALFA		

Docking results showed that gastrodin formed conventional hydrogen bonds with SER350, ASP325 and GLN80 on the p38 protein with a docking score of −3.07 kcal/mol. The nucleoid factor erythroid 2-related factor 2 (NRF2) protein was shown to possess the highest docking score with gastrodin, which is −4.28 kcal/mol. The amino acids present are ARG415, ARG380, SER363, and ASN382 held together by Van der Waals interactions. The docking score of gastrodin with angiotensin-converting enzyme (ACE) is −3.6 kcal/mol, and the amino acids present are PHE330, ARG289, PHE288, and SER286 held together by conventional hydrogen bonds.

Gastrodin also shows binding affinity with the nerve growth factor (NGF) protein, with docking score of −3.48 kcal/mol. The proteins present are THR292 and LEU290, held together by Van der Waals forces. Gastrodin also showed moderate binding affinity towards MAPK, BAX, CX3CR1, MAPK14, and NF-κB with docking scores of −2.99, −2.82, −2.68, −2.65 and −2.45 kcal/mol, respectively. BCL2 showed a lower affinity with a docking score of −1.72 kcal/mol, and the lowest affinity is TNFALFA with a docking score of −1.69 kcal/mol.

### 3.7 ADME study

Gastrodin showed moderate pharmacokinetic properties characterized by decent water solubility (−1.623 log mol/L) but poor membrane permeability, indicated by its low Caco-2 value (0.089 log Papp). The human intestinal absorption is moderate (36.28%), thus suggesting its limited oral bioavailability. The compound’s skin permeability (−2.819 log Kp) is also low, suggesting that there is minimal transdermal absorption. In terms of distribution, gastrodin showed a small volume of distribution (−0.058 log L/kg), indicating there is limited tissue penetration, while its high fraction unbound (0.713 Fu) shows that a large portion remains free in the plasma. However, the compound shows poor blood–brain barrier (−0.93 log BB) and CNS permeability (−3.571 log PS), respectively, therefore suggesting there is minimal central nervous system availability. Metabolically, gastrodin is neither a substrate nor an inhibitor of major CYP450 enzymes (CYP2D6, CYP3A4, CYP1A2, CYP2C19, CYP2C9), indicating a low probability of cytochrome-mediated drug–drug interactions. It shows moderate total clearance (0.172 log ml/min/kg) and is not a substrate for the renal OCT2 transporter, thus suggesting that the involvement of renal secretion is limited. Overall, gastrodin possesses moderate absorption and elimination with minimal metabolic interaction risk, though its poor permeability and BBB penetration may limit its therapeutic potential for CNS-related disorders. The predicted pharmacokinetic properties are summarized in Table 7.

**Table 7:** Predicted pharmacokinetic properties of gastrodin generated from pkCSM.

Pharmacokinetic Properties		Gastrodin
Absorption	Water solubility	−1.623 log mol/L
	Caco2 permeability	0.089 log Papp in 10^-6^cm/s
	Intestinal absorption (human)	36.278 % Absorbed
	Skin permeability	−2.819 log Kp
Distribution	VDss (human)	−0.058 log L/kg
	Fraction unbound (human)	0.713 Fu
	BBB permeability	−0.93 log BB
	CNS permeability	−3.571 log PS
Metabolism	CYP2D6 substrate	No
	CYP3A4 substrate	No
	CYP1A2 inhibitor	No
	CYP2C19 inhibitor	No
	CYP2C9 inhibitor	No
	CYP2D6 inhibitor	No
	CYP3A4 inhibitor	No
Excretion	Total clearance	0.172 log ml/min/kg
	Renal OCT2 substrate	No

### 3.8 Toxicity properties of gastrodin

The toxicity properties of gastrodin, as predicted by pkCSM and ProTox 3.0, indicate low systemic and organ-specific toxicity. According to pkCSM predictions, gastrodin is non-mutagenic and has a low risk of cardiotoxicity. The estimated maximum tolerated dose (MTD) in humans is 0.898 log mg/kg/day. Gastrodin is also predicted to be non-hepatotoxic and non–skin-sensitizing (Table 8). Prediction by ProTox 3.0 showed that gastrodin has an LD_50_ of 3750 mg/kg, with a class 5 toxicity. Table 9 shows the toxicity model report prediction from ProTox 3.0.

**Table 8:** Toxicity properties of gastrodin generated from pkCSM.

Model Name	Gastrodin
AMES TOXICITY	No
Max. tolerated dose (human)	0.898
hERG I inhibitor	No
hERG II inhibitor	No
Oral Rat Acute Toxicity (LD_50_)	1.678
Oral Rat Chronic Toxicity (LOAEL)	3.449
Hepatotoxicity	No
Skin sensitization	No
*T. Pyriformis* toxicity	0.285
Minnow toxicity	4.272

**Table 9:** Toxicity of gastrodin prediction generated from ProTox 3.0.

Classification	Target	Prediction	Probability
Organ toxicity	Hepatotoxicity	Inactive	0.92
	Neurotoxicity	Inactive	0.92
	Nephrotoxicity	Active	0.76
	Respiratory toxicity	Inactive	0.76
	Cardiotoxicity	Inactive	0.64

Toxicity endpoints	Carcinogenicity	Inactive	0.83
	Immunotoxicity	Inactive	0.88
	Mutagenicity	Inactive	0.80
	Cytotoxicity	Inactive	0.86
	BBB-barier	Active	0.94
	Ecotoxicity	Inactive	0.72
	Clinical toxicity	Active	0.51
	Nutritional toxicity	Inactive	0.64

Tox21-Nuclear receptor signalling pathways	Aryl hydrocarbon Receptor (AhR)	Inactive	0.95
	Androgen receptor (AR)	Inactive	0.68
	Androgen Receptor Ligand Binding Domain (AR-LBD)	Inactive	0.76
	Aromatase	Inactive	0.99
	Estrogen Receptor Alpha (ER)	Inactive	0.72
	Estrogen Receptor Ligand Binding Domain (ER-LBD)	Inactive	0.99
	Peroxisome Proliferator Activated Receptor Gamma (PPAR-Gamma)	Inactive	0.99

Tox21-Stress response pathways	Nuclear factor (erythroid-derived 2)-like 2/antioxidant responsive element (nrf/ARE)	Inactive	0.99
	Heat shock factor response element (HSE)	Inactive	0.99
	Mitochondrial Membrane Potential (MMP)	Inactive	0.96
	Phosphoprotein (Tumor Suppressor) p53	Inactive	0.94
	ATPase family AAA domain-containing protein 5 (ATAD5)	Inactive	0.99

Molecular Initiating Events	Thyroid hormone receptor alpha (THRα)	Inactive	0.68
	Thyroid hormone receptor beta (THBβ)	Inactive	0.87
	Transtyretrin (TTB)	Active	0.78
	Byanodine receptor (BYR)	Inactive	0.87
	GABA receptor (GABAR)	Inactive	0.71
	Glutamate N-methyl-D-aspartate receptor (NMDAR)	Inactive	0.94
	Alpha-amino-3-hydroxy-5-methyl-4-isoxazolepropionate receptor (AMPAR)	Inactive	1.0
	Kainate receptor (KAR)	Inactive	1.0
	Acetylcholinesterase (AChE)	Inactive	0.80
	Constitutive androstane receptor (CAR)	Inactive	0.99
	Pregnane X receptor (PXR)	Inactive	0.64
	NADH-quinone oxidoreductase (NADHOX)	Inactive	0.69
	Voltage-gated sodium channel (vgsc)	Inactive	0.92
	Na+/I-symporter (NIS)	Inactive	0.81

Metabolism	Cytochrome CYP1A2	Inactive	0.93
	Cytochrome CYP2C19	Inactive	0.93
	Cytochrome CYP2C9	Inactive	0.75
	Cytochrome CYP2D6	Inactive	0.87
	Cytochrome CYP3A4	Inactive	0.99
	Cytochrome CYP2E1	Inactive	0.96

## 4 Discussion

*Gastrodia elata,* also commonly known as Tianma in traditional Chinese medicine practice, is a member of the Orchidaceae family and has been used for a long time as a traditional herbal medicine to treat neurological disorders. Analysis of the ethanolic extract of *G. elata* of the chemical constituents present in it showed the presence of tannins, alkaloids, saponins, flavonoids, glycosides, phenolic compounds and terpenoids; however, steroids are absent in the extract.

*In silico* prediction was done by using the web-based tool Prediction of Activity Spectra for Substances (PASS) to predict the biological activity spectrum of chemical compounds. To estimate the potential effects and interactions of compounds, the structure–activity relationship is utilized based on their molecular structure. The canonical simplified molecular-input line-entry system (SMILES) format of gastrodin obtained from PubChem was input into the site, and 65 potential biological activities of gastrodin were predicted. Based on the prediction in PASS, several potential activities noted are related to the cardiovascular, respiratory, nervous, immune and hepatic systems.

Gastrodin has shown protective effects on the cardiovascular system and possesses the ability to treat cardiovascular and blood-related ailments. Feng et al.^24^ showed that *G. elata,* which is also known as Tianma, exhibits vasodilatory effects by inhibiting smooth muscle contraction in addition to enhancing the elasticity of the blood vessels and stabilizing the arterial structure. Gastrodin also shows cardioprotective and anti-hypoxic activities, such as protection against hypoxic injury and myocardial ischemia–reperfusion injury, inhibits inflammatory responses in septic cardiac dysfunction and suppresses the expression of cardiac hypertrophy markers.^12^ This further supports the cardioprotective activities of gastrodin, which was predicted. In addition, gastrodin showed a hypotensive effect by vascular relaxation, shown on the thoracic aorta rings of rats through inhibition of the inositol 1,4,5-triphosphate receptor signaling in the sarcoplasmic reticulum.^25^ The vasoprotector properties of gastrodin are shown by its ability to reduce the vascular smooth muscle cells proliferation *in vitro* and suppress neointimal hyperplasia *in vivo*.^12^ The most acute cardiovascular occurrence is due to the major risk factor, atherosclerosis. Studies have found that the development of foam cells in the murine macrophage cells was inhibited by gastrodin, showing its potential anti-atherosclerotic properties.^26^ This further supports the anti-hypercholesterolemic activity prediction of gastrodin. Gastrodin was shown to improve cell proliferation, migration and tube formation in umbilical vein endothelial cells by the activation of the PI3K/Akt signaling pathway and increase the survival rate of flaps, which is linked to the promotion of angiogenesis.^12^, ^27^ This supports the prediction of gastrodin activity to stimulate angiogenesis.

Zhang et al. (2017)^28^ showed that gastrodin is able to inhibit the inflammatory response of LPS-induced acute lung injury and provides protection against it. The gastrodin treatment has shown to significantly reverse the effects induced by LPS, causing an increase in Cdyn and PEF thus, improving lung function. Additionally, gastrodin has been shown to inhibit inflammatory cytokines TNF-α, IL-6 AND IL-1β released in bronchoalveolar lavage fluids (BALF) and NF-κB activation in acute lung injury induced by LPS. Thus, this study supports the prediction that gastrodin acts on the respiratory system.

The accumulation of excess fats in the liver in the absence of alcohol causes nonalcoholic fatty liver disease (NAFLD). Gastrodin is able to improve NAFLD metabolic disorders, hepatic oxidative stress/proinflammatory responses and activates the AMPK/NRF2 pathway. Accumulation of toxic bile acid in the liver will lead to ROS generation, leading to oxidative stress and in turn, causes the inflammatory response.^29^ Gastrodin has shown to be effective in preventing cirrhosis of the liver by its ability to reduce the inflammatory response and block oxidative stress caused by bile duct ligation-induced hepatic fibrosis. It also improves the liver function and reduces the liver injury.^30^ These findings support the prediction that gastrodin is hepatoprotective.

Shu et al. (2013)^31^ showed that gastrodin possesses anticancer immunomodulatory properties by promoting NF-κB mediated gene transcription in CD4 + T cells. Additionally, Liu et al. (2019)^32^ showed that gastrodin is able to stimulate cellular responses and has the potential to be a chemical adjuvant in vaccine development for tumour cells. Li et al. (2023)^33^ reviewed the potential of gastrodin as a therapeutic option in treating COVID-19 and its complications, as gastrodin has shown to inhibit angiotensin II (AngII) which is an important factor in COVID-19. Thus, these findings support the prediction that gastrodin possesses immunoprotective properties.

Other notable predictions by gastrodin, specifically activities on the nervous system, include its role as a GABA aminotransferase inhibitor and a potential treatment for dementia. GABA is a crucial inhibitory neurotransmitter of the nervous system and impairment of GABAergic transmission leads to epileptic seizures. Impairment of GABAergic transmission may be caused by genetic mutations or the use of GABA receptor antagonists. Yang et al. (2021)^34^ showed that gastrodin is able to reduce the severity of seizures and weaken the neuronal excitotoxicity in pilocarpine-induced TLE models. In the nervous system, both astrocytes and microglia worked together to regulate inflammatory responses and has an important role in neuroinflammation and degenerative diseases. A disorder to the nervous system can cause inflammation and glial cell activation which leads to neuronal damage. Gastrodin is able to reduce neuronal apoptosis by reducing the inflammatory responses and oxidative stress, regulates the microglia activation and lowers the damage caused to astrocytes.^35^ Therefore, the prediction for gastrodin and its nervous system activities is supported.

### 4.1 Gas chromatography-mass spectrometry analysis

The ability of gastrodin to easily dissolve in water indicates that it is a polar compound. Gas chromatography-mass spectrometry is able to detect volatile and nonpolar compounds.^36^ The GC–MS analysis of EEGE revealed a total of 17 compounds that may have potential pharmacologic activities. Butane, 1,1-diethoxy-3-methyl- showed the highest probability (92.1), followed by ethane, 1,1-diethoxy- with a probability (89.1). The third-highest probability compound is disulfide, methyl 1-methyl-1-(methylthio)propyl (82.8). Although the probability of butane, 1,1-diethoxy-3-methyl- shows the highest probability, the compound that has the highest peak area (94.019%) is ethane, 1,1-diethoxy- is detected in EEGE. Ethane, 1,1-diethoxy- or also known as acetal, is an ether by nature and a flavouring agent.^37^ Additionally, it possesses anti-seizure activity.^38^ Yang et al. (2021) showed that gastrodin is able to exhibit anti-seizure capabilities in the hippocampus by enhancing GABA_A_ receptor expression.^34^ This observation suggests further investigation into the potential contribution of other constituents, including ethane, 1,1-diethoxy-, to the overall pharmacological and anti-seizure activities of the extract.

### 4.2 Fourier transform infrared spectrometry analysis

FTIR spectra analysis showed similarities and differences in chemical profiles between the gastrodin standard and the ethanolic extract of *G. elata* (EEGE). The results showed both EEGE and the gastrodin standard exhibit absorption peaks at 3222.33 cm^−1^ (hydroxyl stretch), confirming that gastrodin is the active compound in the *G. elata*. The gastrodin standard showed characteristic absorption peaks that indicates its chemical structure, including a carbonyl stretching at 1663.94 cm^−1^, and multiple bands spanning the fingerprint regions (1600–1300 cm^−1^, 1450–1050 cm^−1^, 1080–620 cm^−1^). The ethanolic extract showed absorptions at 3293.78 cm^−1^ (O–H stretch), 2928.10 cm^−1^ (C–H stretch), and 1645.32 cm^−1^ (carbonyl stretch).

### 4.3 Antioxidant level and capacity

The results showed that EEGE has strong antioxidant levels and capacity. Additionally, the PASS prediction also showed that the main bioactive compound, gastrodin, has antioxidative capabilities. Along with the presence of other bioactive compounds such as alkaloids, saponins, flavonoids, glycosides, terpenoids and phenolic compounds, this shows that EEGE is capable of preventing oxidative damage and neutralizing free radicals.

### 4.4 Molecular docking and *in silico* analysis

In the molecular docking studies, gastrodin was docked with ACE, BAX, BCL2, CX3CR1, MAPK 14, MAPK, nerve growth factor (NGF), NF-κB, NRF2, p38, and TNF-α proteins. Docking score results showed that gastrodin has a high affinity for NRF2, ACE, nerve growth factor (NGF) and p38. The results indicate that gastrodin exhibits binding affinity towards p38, a protein associated with the mitogen-activated protein kinase (MAPK) pathway. This pathway plays a pivotal role in regulating various cellular processes, and its dysregulation has been implicated in the development of cardiovascular disorders, inflammation, cancer, and other diseases. Additionally, gastrodin demonstrates a similarly strong binding affinity towards nucleoid factor erythroid 2-related factor 2 (NRF2), a key transcription factor that protects cells against oxidative stress and neurodegenerative diseases. Gastrodin has shown to be able to reduce the apoptosis and cellular dysfunction induced by *tert*-butyl hydroperoxide (TBHP) by protecting the HUVECs from TBHP-induced cellular apoptosis by activating the nuclear factor (erythroid-derived 2)-like 2 (NRF2)/heme oxygenase 1 (HO-1) pathway.^39^ Therefore, gastrodin emerges as a promising therapeutic candidate, as it effectively targets two critical cellular pathways involved in the pathogenesis of life-threatening diseases.

The results also show that gastrodin has a strong binding affinity towards the ACE, which is an important part of the renin-angiotensin system. It is mainly responsible for the conversion of angiotensin I to angiotensin II and breaks down bradykinin. ACE is vital for the maintenance of normal blood pressure and electrolyte balance by the homeostatic mechanism in mammals.^40^ The docking score of gastrodin with ACE is −3.6, and the amino acids present are PHE330, ARG289, PHE288, and SER286 held together by conventional hydrogen bonds. ACE (angiotensin converting enzyme) is responsible for converting angiotensin I to angiotensin II whereby the latter is responsible for elevating blood pressure by acting as a vasoconstrictor. Gastrodin is able to target and inhibit Ang II, aldosterone (ALD), ACE and ATIR thus improving blood pressure.^41^

Gastrodin showed a high binding score of −3.48 kcal/mol towards nerve growth factor (NGF) protein, indicating that it has potential effects on the nervous system. Yang et al. (2021) assessed gastrodin’s potential as an anticonvulsant by administering it to epileptic rat models induced by lithium-pilocarpine. The results showed that gastrodin inhibits the levels of BDNF and NGF, and activates the AMPK/PPARα signal transduction pathway, thereby reducing the onset of pediatric epilepsy.^42^

### 4.5 ADME and toxicity study of gastrodin

The BBB permeability is influenced heavily by the nature of the molecules. Micromolecules with molecular weight of less than 600 Da, chain length of less than 6 amino acids and lipid soluble molecules are able to easily cross the BBB.^43^ Gastrodin is fairly soluble in water but has low BBB permeability due to its hydrophilic nature.^44^ Lin et al (2007) reported that the exposure of gastrodin in the brain is small and is possibly due to the rapid metabolism of gastrodin into HBA.^2^, ^45^ Due to the hydrophilic nature of gastrodin and its rapid metabolism, it is a challenge to permeate the BBB and exhibit its effects onto the CNS. Therefore to overcome this, Luo et al (2024) used microfluidic technology to prepare multivesicular liposomes (MVLs) containing gastrodin for oral delivery.^46^ Gastrodin, a phenolic glycoside, is primarily hydrophilic in nature, yet it demonstrates notable central nervous system (CNS) activity. Despite the general limitation of hydrophilic compounds in crossing the blood–brain barrier (BBB), gastrodin’s effectiveness can be explained by two key mechanisms. First, it is capable of reaching the CNS shortly after intravenous or oral administration via glucose transporter (GLUT) systems,^47^ which facilitate the movement of hydrophilic molecules and contribute to achieving an effective peak plasma concentration (Cmax). Second, gastrodin is metabolized into p-hydroxybenzyl alcohol, its active aglycone metabolite. This metabolite has a lower molecular weight and increased lipophilicity compared to gastrodin, enabling it to cross the BBB more efficiently. Thus, while gastrodin ensures good solubility and systemic absorption, its metabolite enhances CNS penetration. Together, these complementary properties make gastrodin an effective neuroactive compound despite its glycosidic and hydrophilic nature. Compounds that have an absorbance of below 30% is considered to be poorly absorbed.^23^ Although gastrodin is said to be rapidly absorbed by the intestinal tract, it is found to be influenced by intestinal microbiota according to a study conducted by Nepal et al.^48^ Thus, leading to the prediction by pkCSM on the human intestinal absorption for gastrodin to be moderate. As gastrodin does not permeate the skin, this implies that it is not absorbed by the dermal route. Gastrodin was shown to have a limited volume of distribution, indicating limited tissue penetration. Despite the low volume of distribution, gastrodin is shown to be distributed mainly in the kidneys. Other organs involved are the heart, liver and lung with moderate distribution, and a small fraction of distribution is found in the brain and spleen, and these two organs showed to have the lowest distribution of gastrodin found.^45^, ^49^ Gastrodin has also shown to be unreactive towards the substrates and inhibitors of CYP450 enzymes, consistent with prediction by ProTox 3.0. Additionally, it is readily metabolized into many different metabolites after the biotransformation process.^50^, ^51^

ADMET results predicted from pkCSM and ProTox 3.0 showed that gastrodin is an extremely safe compound and does not cause severe organ toxicity, as it shows inactivity toward hepatotoxicity, neurotoxicity, respiratory toxicity and cardiotoxicity. This suggests that the likelihood of adverse effects on the vital organs is low. On the other hand, nephrotoxicity for gastrodin was predicted to be active. The toxicity endpoints of gastrodin were predicted to be inactive as well for carcinogenicity, mutagenicity, cytotoxicity and immunotoxicity. This shows that gastrodin does not contribute any genotoxic or immunotoxic risk. The prediction of BBB-barrier toxicity is active. According to Pires et al.,^23^ it is difficult to measure the blood–brain permeability and a more direct measurement is based on blood–brain permeability-surface area products, which is obtained from *in situ* brain perfusions after the injection of a compound directly into the carotid artery. Although pkCSM predicted poor blood–brain barrier (BBB) permeability, ProTox 3.0 identified BBB-related activity; therefore, further experimental studies are required to clarify gastrodin's BBB penetration and CNS exposure. Gastrodin also showed inactivity toward disruption of the endocrine system, as it shows inactivity for all the tested receptors such as AhR, AR, ER, PPAR-γ, and aromatase. Additionally, the inactivity of gastrodin towards the important molecular targets, including neurotransmitter receptors (GABA, NMDA, AMPA, KAR), acetylcholinesterase, voltage-gated sodium channels, and nuclear receptors (CAR, PXR, THRα/β), suggests that gastrodin does not disrupt important cellular processes. On the other hand, the active binding of gastrodin towards transthyretin (TTB) showed that there may be some influence on the thyroid hormone transport or related signalling mechanisms. The affinity of gastrodin towards prostaglandin G/H synthase 1 (PGH1) indicates the potential role of gastrodin in inflammatory responses, as it was traditionally used to treat inflammation and possesses anti-inflammatory properties. Gastrodin also possess high LD_50_ of 3750 mg/kg, indicating that it does not cause adverse effects at high doses.

## 5 Conclusion

Overall, PASS predictions support gastrodin as a promising bioactive compound with diverse pharmacological actions applicable to cardiovascular, metabolic, neurological, and immunological disorders. Gas chromatography-mass spectrometry analysis of the possible bioactive compounds in EEGE showed that it possesses potential pharmacological activities. FTIR analysis comparing the gastrodin standard and the EEGE confirms that the active compound in EEGE is gastrodin. Molecular docking analysis showed that gastrodin interacts with important proteins responsible for regulating important cellular pathways, which are responsible for many life-threatening diseases. The use of pkCSM and ProTox 3.0 to predict the pharmacokinetics and toxicity suggests that gastrodin is safe and well-tolerated, as it has a relatively high LD_50_. It also has low potential for genetic, hepatic, cardiac, dermal and systemic toxicity. On the other hand, gastrodin is predicted to potentially cause nephrotoxicity and possess BBB-related effects. It binds well with PGH1, indicating a potential role in prostaglandin-mediated inflammatory and central nervous system pathways. Further validation of the docking results through molecular dynamics simulations, including RMSD, RMSF, radius of gyration, hydrogen bond analysis, and binding free energy calculations over an extended simulation period (≥20 ns), will be carried out in future studies to confirm the stability and dynamic behaviour of the identified complexes.

## CRediT authorship contribution statement

**Jia Jun Chuah:** Writing – original draft; Writing – review & editing; Methodology; Investigation; Formal analysis; Visualization. **Morris Jehssica Shri:** Writing – original draft; Writing – review & editing; Methodology; Investigation; Formal analysis. **Balakrishnan Shanthakumar:** Conceptualization; Software; Validation; Data curation; Formal analysis. **Krishnamoorthy Venkateskumar:** Supervision; Writing – review & editing. **Karupiah Sundram:** Validation; Supervision; Writing – review & editing. **Subramani Parasuraman:** Conceptualization; Validation; Resources; Data curation; Supervision; Writing – review & editing; Visualization; Project administration.

## Declaration of competing interest

The authors declare that they have no known competing financial interests or personal relationships that could have appeared to influence the work reported in this paper.
